# Pathological Sequelae Associated with Skeletal Muscle Atrophy and Histopathology in G93A*SOD1 Mice

**DOI:** 10.3390/muscles2010006

**Published:** 2023-02-02

**Authors:** Richa Aishwarya, Chowdhury S. Abdullah, Naznin Sultana Remex, Sadia Nitu, Brandon Hartman, Judy King, Mohammad Alfrad Nobel Bhuiyan, Oren Rom, Sumitra Miriyala, Manikandan Panchatcharam, A. Wayne Orr, Christopher G. Kevil, Md. Shenuarin Bhuiyan

**Affiliations:** 1 Department of Pathology and Translational Pathobiology, Louisiana State University Health Sciences Center at Shreveport, Shreveport, LA 71103, USA; 2 Department of Molecular and Cellular Physiology, Louisiana State University Health Sciences Center at Shreveport, Shreveport, LA 71103, USA; 3 Department of Medicine, Louisiana State University Health Sciences Center at Shreveport, Shreveport, LA 71103, USA; 4 Department of Cellular Biology and Anatomy, Louisiana State University Health Sciences Center at Shreveport, Shreveport, LA 71103, USA

**Keywords:** skeletal muscle, ALS, G93A*SOD1, histopathology, mitochondria

## Abstract

Amyotrophic lateral sclerosis (ALS) is a complex systemic disease that primarily involves motor neuron dysfunction and skeletal muscle atrophy. One commonly used mouse model to study ALS was generated by transgenic expression of a mutant form of human superoxide dismutase 1 (SOD1) gene harboring a single amino acid substitution of glycine to alanine at codon 93 (G93A*SOD1). Although mutant-SOD1 is ubiquitously expressed in G93A*SOD1 mice, a detailed analysis of the skeletal muscle expression pattern of the mutant protein and the resultant muscle pathology were never performed. Using different skeletal muscles isolated from G93A*SOD1 mice, we extensively characterized the pathological sequelae of histological, molecular, ultrastructural, and biochemical alterations. Muscle atrophy in G93A*SOD1 mice was associated with increased and differential expression of mutant-SOD1 across myofibers and increased MuRF1 protein level. In addition, high collagen deposition and myopathic changes sections accompanied the reduced muscle strength in the G93A*SOD1 mice. Furthermore, all the muscles in G93A*SOD1 mice showed altered protein levels associated with different signaling pathways, including inflammation, mitochondrial membrane transport, mitochondrial lipid uptake, and antioxidant enzymes. In addition, the mutant-SOD1 protein was found in the mitochondrial fraction in the muscles from G93A*SOD1 mice, which was accompanied by vacuolized and abnormal mitochondria, altered OXPHOS and PDH complex protein levels, and defects in mitochondrial respiration. Overall, we reported the pathological sequelae observed in the skeletal muscles of G93A*SOD1 mice resulting from the whole-body mutant-SOD1 protein expression.

## 1. Introduction

Amyotrophic lateral sclerosis (ALS) is a fatal neuromuscular disease having an annual prevalence of about 4 to 6 per 100,000 people with a median age of onset between 51 and 66 years [1]. ALS typically has a focal onset primarily affecting the motor neurons (MNs), resulting in progressive upper and lower MNs degeneration and death, muscle atrophy, distress of the respiratory system, and accumulation of cellular protein aggregates [2]. The reported clinical manifestation of ALS had high variability in the onset’s age and site, the onset’s body region, relative upper versus lower MN involvement, disease progression, and the development of frontotemporal dementia [3]. Progressive muscle weakness is the hallmark of ALS clinical manifestation, together with muscle atrophy, fasciculation, muscle cramps, and muscle stiffness [4]. The usual clinical presentation of ALS disease reported is unilateral distal muscle weakness and atrophy in upper or lower limb muscles (spinal ALS) or bulbar muscles (bulbar ALS). Muscle weakness is typically accompanied by hyperreflexia, muscle wasting, and fasciculations. Rare disease presentations in some ALS patients reported weakness in the respiratory or axial muscles. The degenerative process, in most cases, extends to the frontal and anterior temporal lobe, giving rise to a variable degree of executive dysfunction. Due to multifocal disease processes, ALS is clinically recognized as a heterogeneous syndrome with distinct motor and extra-motor manifestations. Currently, available drugs for the treatments of ALS, including Riluzole (glutamate antagonist) [5] and Edaravone (free radical scavenger) [6], have shown little efficacy in slowing down the disease progression and are unable to reverse motor neuron damage and muscle weakness. Therefore, there is a critical need to elucidate the molecular mechanisms of the development of ALS pathobiology to identify potential therapeutic targets.

A small number of identified cases of ALS patients are genetically inherited (approximately 10%), and the remaining majority of the cases are sporadic. Among these sporadic cases, only 5–10% of cases were identified as having causative mutations in genes [7]. A large number of the identified cases of familial ALS (15–20%) were reported to have mutations in the gene encoding SOD1, and more than 45 mutations have been identified in SOD1 linking to familial ALS [8]. Multiple lines of ALS-associated SOD1 mutants (G93A, G37R, and G85R) overexpressing mice models were developed to reminiscent human ALS to determine the disease mechanisms and cellular pathobiology [9–13]. Despite extensive research using genetic mice models, the etiology of ALS pathology remained elusive, and the cellular targets critical to the ALS disease process remained unknown. Most of the reported mice models were generated by using several human mutations in SOD1, where whole-body expression of these mutant SOD1 leads to a pathology reminiscent of human ALS characterized by limb weakness, muscle wasting, and paralysis [9–13]. However, despite the reports on the expression of mutant SOD1 in the skeletal muscle, it remained unknown whether skeletal muscle resident SOD1 directly contributes to any pathological sign of ALS.

Several organ-specific transgenic mice models of ALS had been generated to recapitulate the human disease by constitutively expressing several of the human mutant-SOD1 (e.g., G93A and G85R) in postnatal mouse neurons to dissect the role of MN in the development of ALS pathobiology. Surprisingly, these neuron-specific transgenic mice having high-level expression and accumulation of the mutant SOD1 proteins didn’t recapitulate the human pathology and failed to produce any detectable sign of MN pathology [14]. Furthermore, these mice showed no changes in hindlimb muscle strength, signs of muscle denervation, or motor neuron disease. Surprisingly, duplicating the mutant SOD1 load (G93A*SOD1) in MNs by using a double-transgenic mouse (hMg-SOD1^G93A^/Thy1-SOD1^G93A^) did not affect disease onset, disease progression, and MN pathology between the single hMg-SOD1^G93A^ and double-transgenic mouse (hMg-SOD1^G93A^/Thy1-SOD1^G93A^) [14]. Similarly, restricted expression of another mutant SOD1 (SOD1^G86R^) in astrocytes results in astrocytosis without causing MN death or motor dysfunction in vivo [15]. All these mice models developed by using selective expression of mutated SOD1 in either MNs or astrocytes failed to reproduce the ALS-like pathology in mice. Interestingly, muscle-restricted expression of the mutant *SOD1* using a muscle-specific promoter (*MLC/SOD1*^*G93A*^) induced severe muscle atrophy reduced tetanic and specific force, displayed NMJ abnormalities, and developed MN distal axonopathy [16–18]. Severe muscle atrophy in these mice was associated with a significant reduction in muscle strength, sarcomere disorganization, altered mitochondrial morphology and function, and sarcotubular system disorganization [16–18]. Subsequent studies also suggested that muscle atrophy in *MLC/SOD1*^*G93A*^ mice was primarily associated with the toxic effects of the mutant protein in the muscle. These studies also suggest that the MN degeneration in *MLC/SOD1*^*G93A*^ mice may further exacerbate the atrophic phenotype promoting paralysis and muscle wasting as a later event [16,17].

Extensive research over the past decades to delineate the extraordinarily complex ALS disease mechanisms extensively used the global G93A*SOD1 overexpressing mice. G93A*SOD1 mice developed comprehensive disease phenotypes of neurologic, anatomic, and histopathologic defects reminiscent of human ALS. However, despite the skeletal muscle pathology reported in these mutant SOD1 mice, detailed and comprehensive histopathological alterations in the skeletal muscle were never studied in these mice. In the present study, we extensively characterized the pathological sequelae of histological, molecular, ultrastructural, and biochemical alterations in gastrocnemius (Gastro), quadriceps (Quad), tibialis anterior (TA), extensor digitorum longus (EDL), and soleus (Sol) muscles of the G93A*SOD1 mouse. This study demonstrated that muscle pathology in G93A*SOD1 mice was associated with fibrotic remodeling, histopathology, altered myofiber size, abnormal mitochondria, defective mitochondrial respiration, and reduced muscle functions.

## 2. Results

### 2.1. Skeletal Muscles of G93A*SOD1 Mice Showed Atrophy and a Differential Pattern of Mutant SOD1 Protein Expression

**Figure 1.**
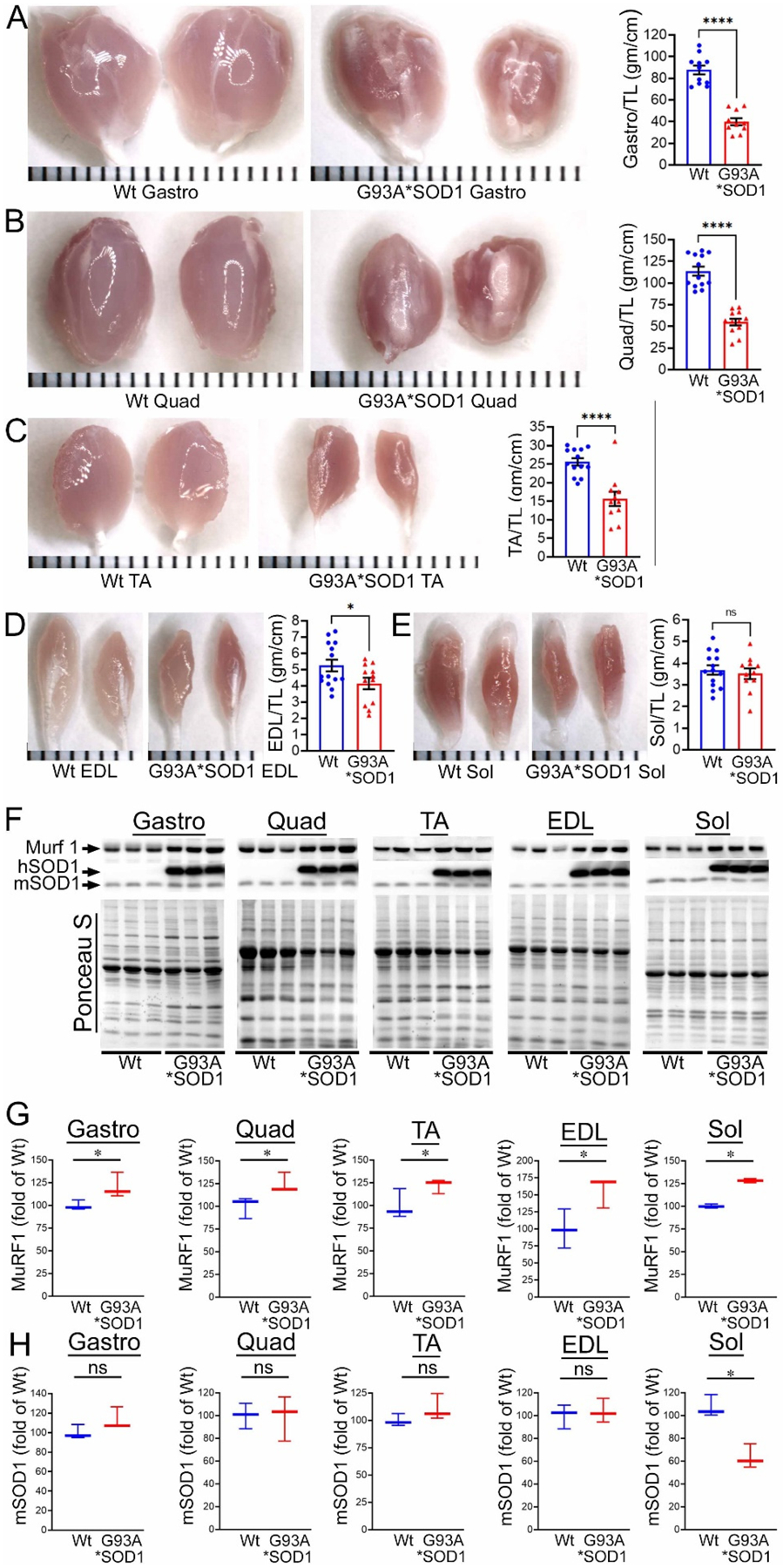
Skeletal muscle mass and mutant-protein level in G93A*SOD1 mice. (**A**–**E**) Representative morphological images and quantifications of muscle weight-to-tibia length (MW/TL) ratio of (**A**) Gastrocnemius (Gastro), (**B**) Quadricep (Quad), (**C**) Tibialis Anterior (TA), (**D**) Extensor digitorum longus (EDL), and (**E**) Soleus (Sol) muscle. Dots in the bar graphs represent individual quantified values for each Wt and G93A*SOD1 mice (n = 6 mice/group). Data are expressed as mean ± SEM. Scale bars: 1 mm for each small bar across X-axes. (**F**) Representative Western blot images of human mutant-SOD1 (hSOD1), endogenous SOD1 (mSOD1), and MuRF1 protein level in muscle tissue lysates of Wt and G93A*SOD1 mice (n = 3 mice/group). Box plots showing the Quantification of the MuRF1 (**G**) and mSOD1 (**H**) protein levels in the muscles of Wt and G93A*SOD1 mice (n = 3 mice/group). Ponceau S staining of the transfer membranes was used to confirm the approximately equal loading and transfer across the gel. Boxes represent interquartile ranges, lines represent medians, and whiskers represent ranges. *p* < 0.05 was considered statistically significant and was determined by Kruskal–Wallis test. * *p* < 0.05, **** *p* < 0.0001, and ns = non-significant. Wt = Wildtype.

**Figure 2.**
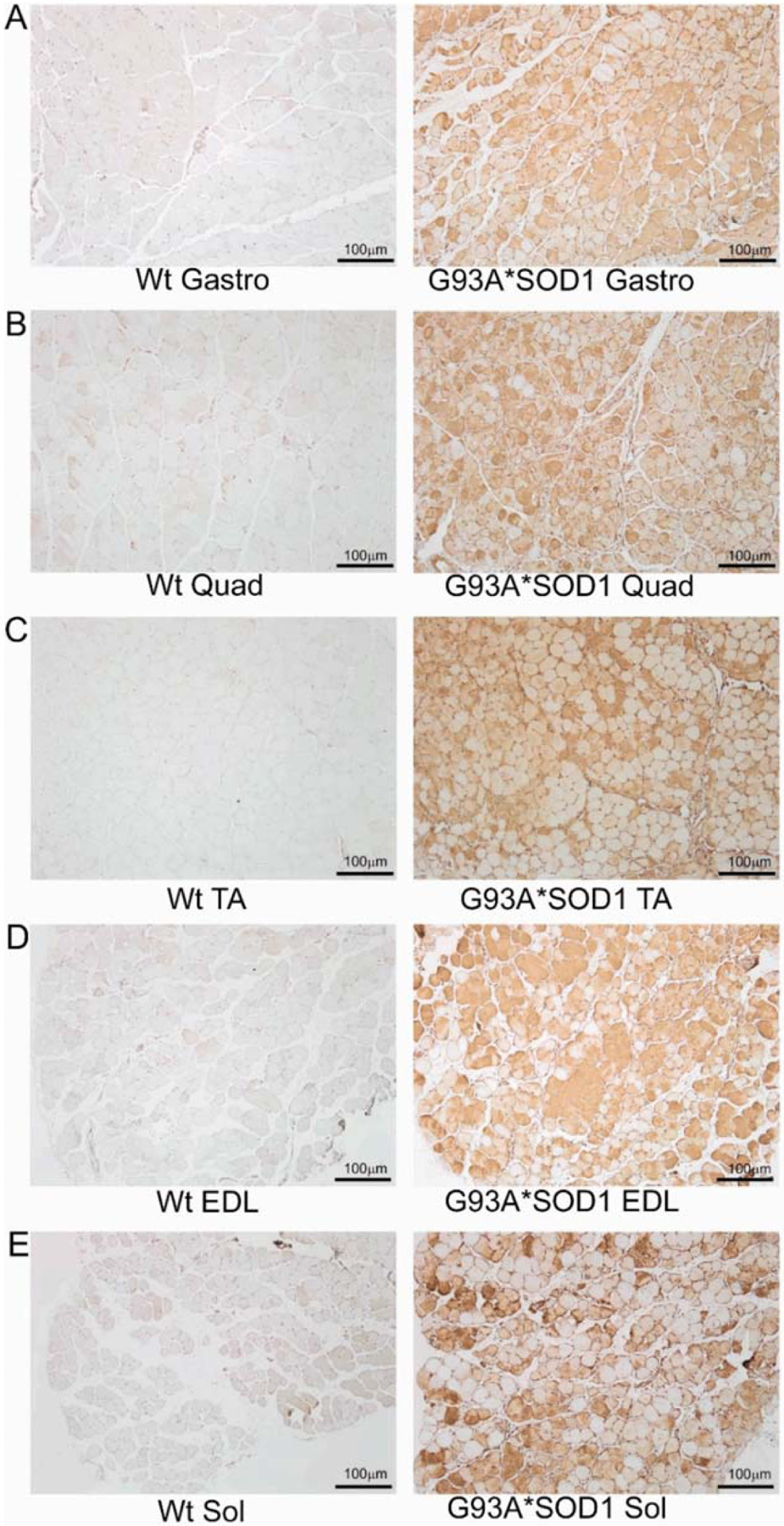
IHC staining of human mutant-SOD1 protein in the muscles of G93A*SOD1 mice. Representative IHC stained images showing the human mutant-SOD1 protein (brown) in the muscle cross-sections of Wt and G93A*SOD1 mice. (**A**) Gastrocnemius (Gastro), (**B**) Quadriceps (Quad), (**C**) Tibialis Anterior (TA), (**D**) Extensor digitorum longus (EDL), and (**E**) Soleus (Sol) muscle. Hematoxylin (blue) was used to counterstain the nuclei. (n = 4 mice/group). Scale bars: 100 μm. Wt = Wildtype.

To investigate the skeletal muscle pathological sequelae associated with the whole-body expression of human mutant-SOD1, we performed a morphometric analysis of the Gastro, Quad, TA, EDL, and Sol muscles of age-matched Wt and G93A*SOD1 mice. Measurements of muscle weight-to-tibia length ratio in freshly isolated skeletal muscles of G93A*SOD1 mice showed reduced muscle mass compared to Wt mice (Figure 1A–E). As all the muscles showed atrophy, we measured the protein level of the E3 ligase MuRF1 in these muscles as a marker of muscle atrophy. Western blot analysis showed increased MuRF1 protein levels in all the muscles of G93A*SOD1 mice (Figure 1F,G). Extensive research over the years using rodent models developed by mutant SOD1 overexpression demonstrated that the mutant protein’s toxicity depends on the mutant action within different cells determining the disease’s onset and progression. The mutant SOD1 accumulation has been a hallmark feature of ALS pathology in mutant-SOD1-expressing animal models. We observed the mutant human SOD1 protein level in all skeletal muscles isolated from G93A*SOD1 mice confirming the ubiquitous expression of mutant human SOD1 in this mouse model of ALS (Figure 1F,H). Increased expression of the exogenous mutant human SOD1 protein in the skeletal muscle of G93A*SOD1 mice did not significantly alter the endogenous mouse SOD1 level in Gastro, Quad, TA, EDL, and Sol muscles. We also confirmed the expression of the mutant human SOD1 protein in all the muscle fibers using IHC staining with anti- SOD1 antibody specific for the human SOD1 (Figure 2A–E). Interestingly, the mutant SOD1 protein level throughout the skeletal muscle was not uniform and showed a differential pattern. Overall, G93A*SOD1 mice showed ubiquitous and nonuniform expression patterns of human mutant-SOD1, skeletal muscle atrophy, and an increased level of the atrophy marker (MuRF1) in the skeletal muscle.

### 2.2. Reduced Endurance and Tolerance to Exercise in G93A*SOD1 Mice

**Figure 3.**
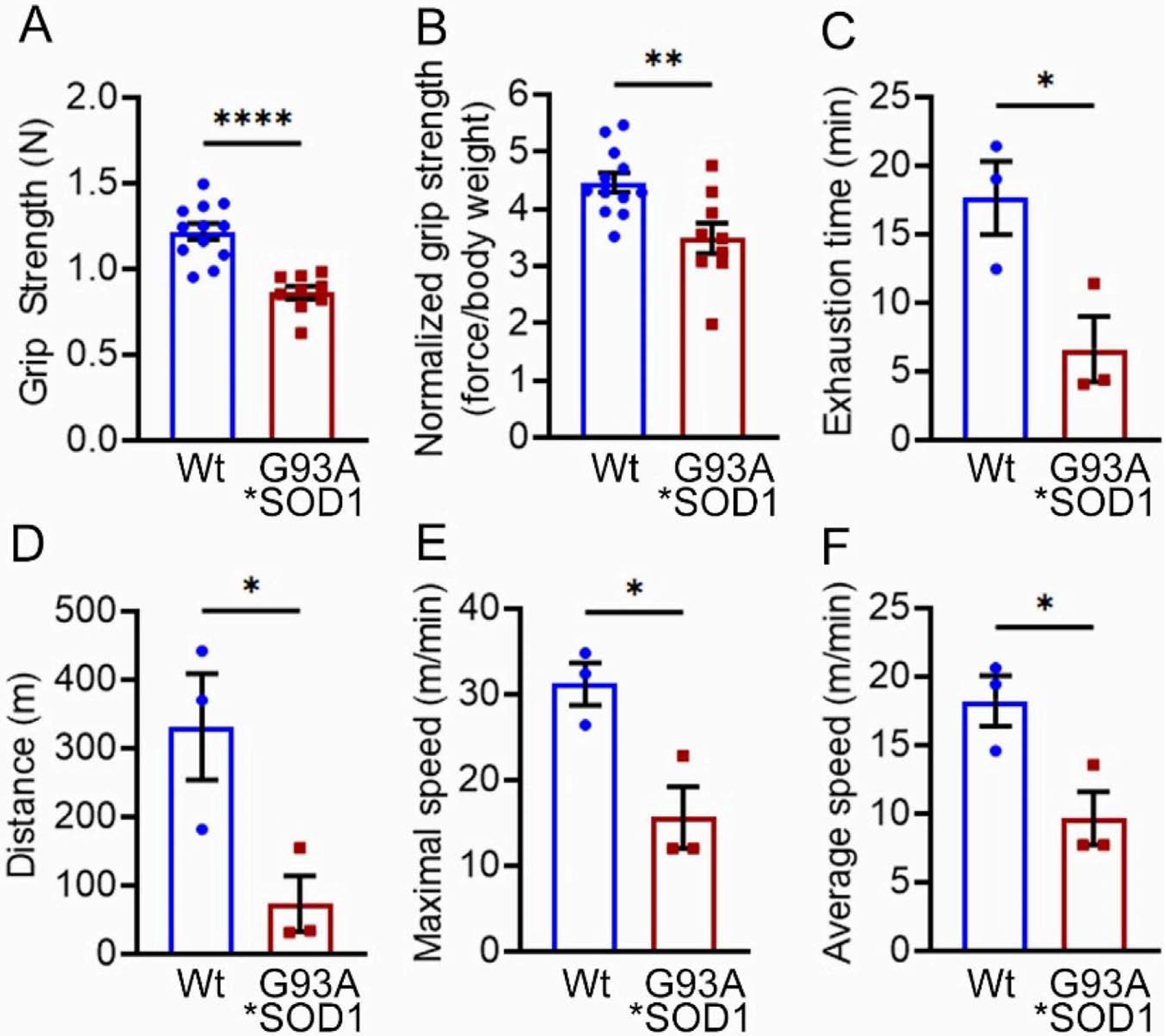
G93A*SOD1 mice showed reduced endurance and tolerance to exercise. Comparison of (**A**) absolute grip strength (N) and (**B**) grip strength normalized to body weight (force/body weight) between Wt and G93A*SOD1 mice (n = 12 mice/group). Exercise tolerance test parameters showing (**C**) time to exhaustion (min), (**D**) maximum running distance (meters), (**E**) maximum speed attained (meters/min), and (**F**) average speed (meters/min) in Wt and G93A*SOD1 mice (n = 3 mice/group). Bars represent Mean ± SEM, and the dots in the bars represent data for each mouse. *p* < 0.05 between groups was considered statistically significant and was determined by unpaired Student’s *t*-test. * *p* < 0.05, ** *p* < 0.01, and **** *p* < 0.0001. Wt = Wildtype.

Skeletal muscle functional assessment in the mice was performed by grip strength measurement and graded maximal exercise testing. Measurements of absolute forelimb grip strength (Figure 3A) and forelimb grip strength normalized to body weight (Figure 3B) showed reduced physical endurance in G93A*SOD1 mice compared to Wt mice. We also performed an exercise tolerance test using a forced treadmill running. Interestingly, G93A*SOD1 mice showed compromised exercise capacity to run on a treadmill compared to Wt mice, indicated by exercise tolerance test parameters including reduced exhaustion time (Figure 3C), maximum distance run (Figure 3D), maximal speed (Figure 3E), and average speed (Figure 3F). Overall, G93A*SOD1 mice showed reduced endurance and tolerance to exercise.

### 2.3. Skeletal Muscles Collagen Deposition and Myopathy in G93A*SOD1 Mice

**Figure 4.**
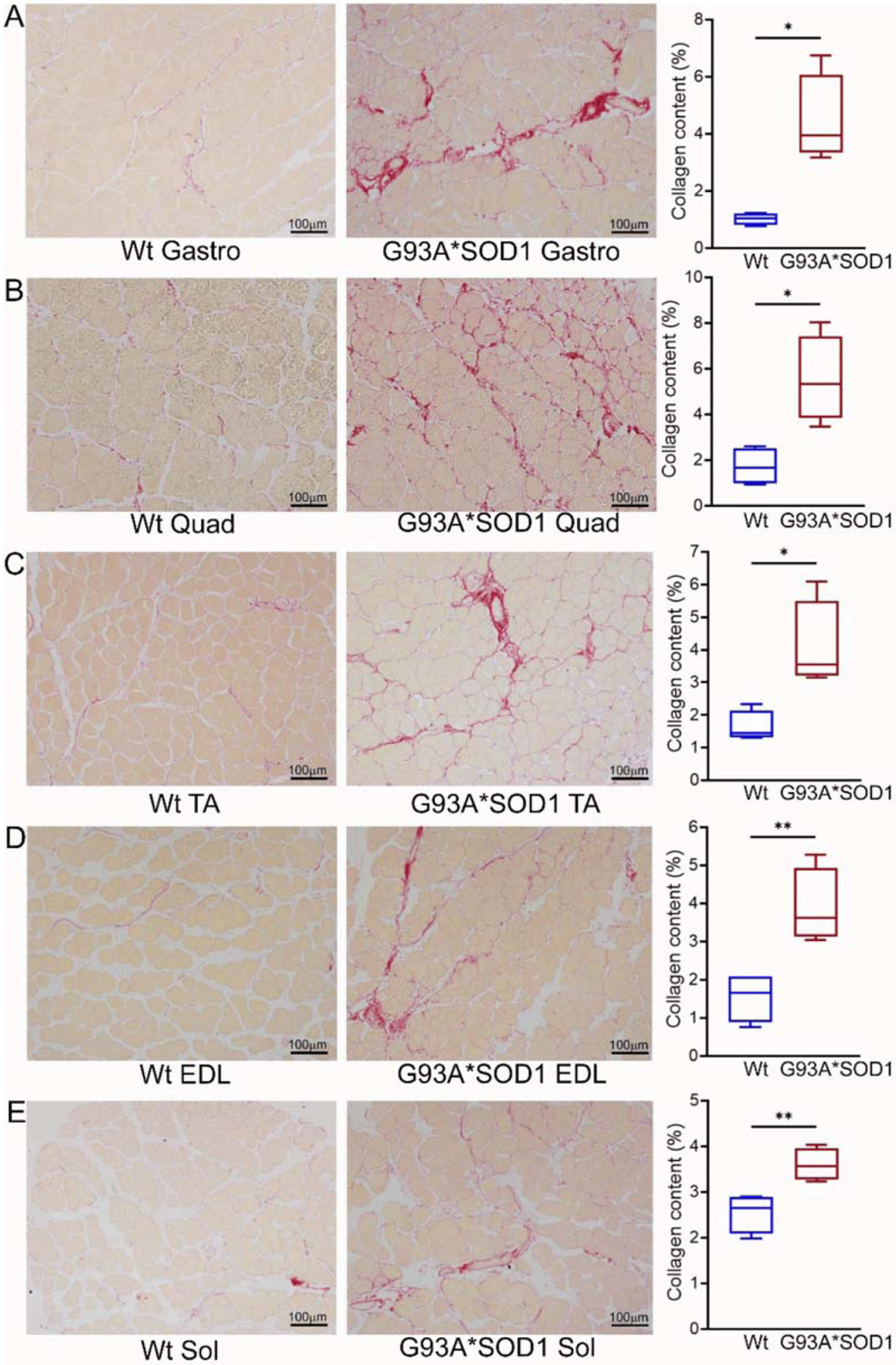
Increased collagen deposition in the skeletal muscles of G93A*SOD1 mice. Representative Picro-Sirius red (PSR) stained images of (**A**) Gastrocnemius (Gastro), (**B**) Quadriceps (Quad), (**C**) Tibialis Anterior (TA), (**D**) Extensor digitorum longus (EDL), and (**E**) Soleus (Sol) muscle. Scale bars: 100 μm. (**A**–**E**) The box plots represent the quantified percent collagen content in the skeletal muscles Wt and G93A*SOD1 mice (n = 4 mice/group). Boxes represent interquartile ranges, lines represent medians, and whiskers represent ranges. *p* < 0.05 between groups was considered statistically significant and was determined by unpaired Student’s *t*-test. * *p* < 0.05 and ** *p* < 0.01. Wt = Wildtype.

Several studies using animal models and human samples suggest fibrotic remodeling occurs in different organs in ALS [19]. However, histopathological characterization of the skeletal muscles of mouse models of ALS is scarce and remains understudied. Therefore, we evaluated fibrotic remodeling in the skeletal muscles (Gastro, Quad, TA, Sol, and EDL muscles) isolated from Wt and G93A*SOD1 mice using Picro-Sirius Red (PSR) staining (Figure 4A–E). All five skeletal muscles from G93A*SOD1 mice exhibited significantly increased interstitial and perivascular collagen deposition compared to Wt mice. Therefore, the fibrotic remodeling of the muscles accompanied skeletal muscle atrophy in the G93A*SOD1 mice.

**Figure 5.**
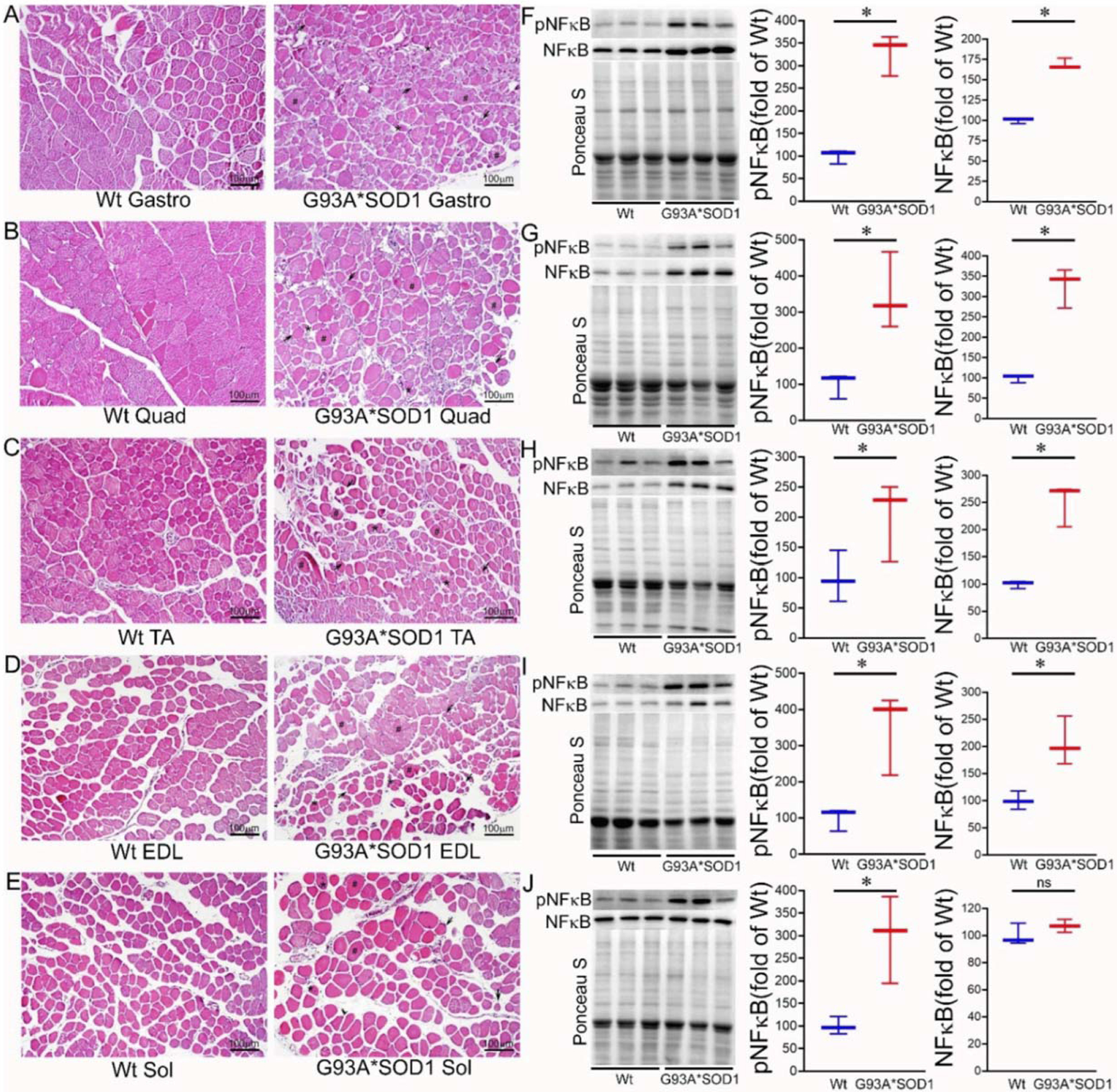
H&E staining of skeletal muscle sections of Wt and G93A*SOD1 mice. Representative images of H&E stained cross-sections muscle sections of (**A**) Gastrocnemius (Gastro), (**B**) Quadriceps (Quad), (**C**) Tibialis Anterior (TA), (**D**) Extensor digitorum longus (EDL), and (**E**) Soleus (Sol) muscle. G93A*SOD1 mice muscles exhibit myopathic changes indicated by markedly increased variation in fiber size, including hypertrophic fibers (#), atrophic fibers (*), and endomysial fibrosis (black arrows) compared to Wt mice muscle sections (n = 4 mice/group). Scale bars: 100 μm. (**F**–**J**) Representative Western blot images and densitometric quantification showing pNFκB and NFκB protein levels in Gastro, Quad, TA, EDL, and Sol muscles. Ponceau S staining of the transfer membranes was used to confirm the approximately equal loading and transfer across the gel. Box plots showing the protein levels of pNFκB and NFκB in Gastro, Quad, TA, EDL, and Sol muscles (n = 3 mice/group). Boxes represent interquartile ranges, lines represent medians, and whiskers represent ranges. *p* < 0.05 between groups was considered statistically significant and was determined by the non-parametric Kruskal–Wallis test. * *p* < 0.05, ns = non-significant. Wt = Wildtype.

Extensive research demonstrated a link between the activation of inflammatory responses and fibrotic remodeling [20,21]. Therefore, we performed the histological analysis of the skeletal muscles (Gastro, Quad, TA, EDL, and Sol) isolated from Wt and G93A*SOD1 mice by hematoxylin and eosin (H&E) staining. H&E stained muscle sections in G93A*SOD1 mice showed chronic myopathic changes, including atrophic fibers, fiber size variation, and endomysial fibrosis compared to Wt muscle sections (Figure 5A–E). H&E staining also showed an increased interstitial eosinophilic collagen deposition in G93A*SOD1 mice muscle sections compared to that in Wt mice muscle sections. In addition, all five skeletal muscles in G93A*SOD1 mice also showed a substantial increase in interstitial nuclei located surrounding the muscle fibers at the perimysium and around individual fibers in the endomysium.

Emerging evidence suggests a direct link between NF-κB activation and muscle mass loss under various pathophysiological conditions. Muscle-specific activation of NF-κB was also reported to cause profound muscle wasting by augmenting the expression of MuRF1 [22]. Therefore, we measured the protein level of NF-κB, which is a pivotal mediator of inflammatory responses in the different tissues [19]. Western blot analysis showed an increased phosphoNF-κB level in all the skeletal muscles (Gastro, Quad, TA, EDL, and Sol) isolated from G93A*SOD1 mice suggesting activation of inflammation in these muscles (Figure 5F–J).

**Figure 6.**
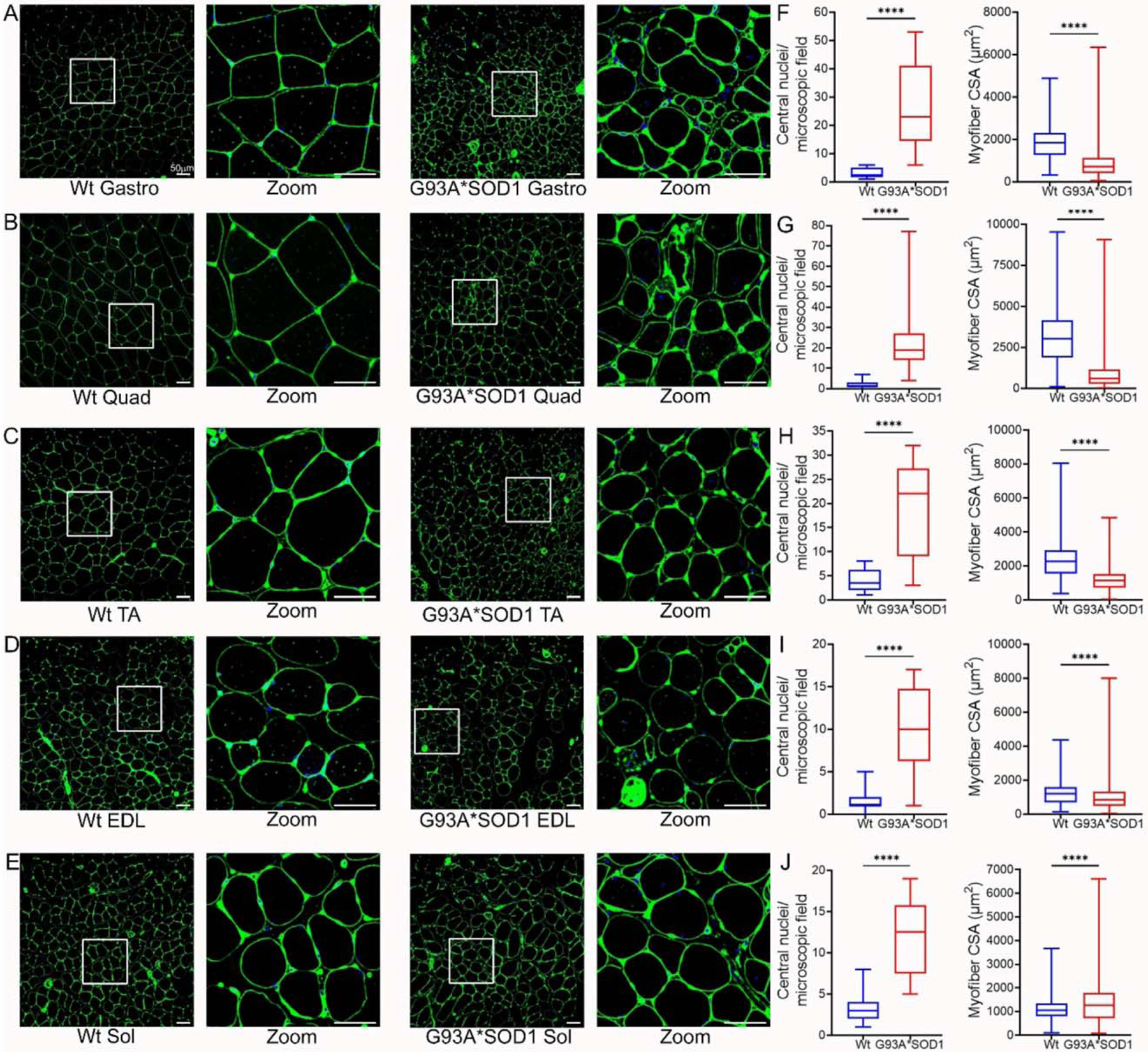
Variable myofiber size and central nuclei presence in G93A*SOD1 muscles. Representative WGA stained (green) images of (**A**) Gastrocnemius (Gastro), (**B**) Quadriceps (Quad), (**C**) Tibialis Anterior (TA), (**D**) Extensor digitorum longus (EDL), and (**E**) Soleus (Sol) muscle sections (Wt and G93A*SOD1 mice). A white rectangle box indicated digitally magnified areas indicating the increased presence of central nuclei (white arrows) in G93A*SOD1 mice myocytes. Scale bars: 50 μm. Box plots represent the central nuclei per microscopic field (10–40 microscopic fields from n = 4 mice per group for each muscle) and average myocyte CSA (μm^2^) (2000–5000 cells from n = 4 mice/group) for (**F**) Gastro, (**G**) Quad, (**H**) TA, (**I**) EDL, and (**J**) Sol muscle sections from Wt and G93A*SOD1 mice, respectively. Boxes represent interquartile ranges, lines represent medians, and whiskers represent ranges. P values were determined by unpaired Student’s *t*-test. **** *p* < 0.0001; Wt = Wildtype.

To complement the findings of H&E staining, we also performed wheat germ agglutinin (WGA) staining to delineate the myofiber areas. WGA staining of the skeletal muscle sections showed heterogeneous fiber size and the presence of myofiber with central nuclei in the G93A*SOD1 mice (Figure 6A–E). Several different myopathies, developmental abnormalities, or muscle regeneration were associated with the extent of central nuclei in skeletal muscles [23–26]. In addition, G93A*SOD1 mice showed increased myofiber central nuclei and significant changes in fiber size in all muscles compared to Wt muscles (Figure 6F–J). Overall, the skeletal muscle fibers isolated from G93A*SOD1 mice showed increased fibrotic remodeling, fiber size variation, and signs of activation of inflammatory pathways.

### 2.4. Skeletal Muscles Isolated from G93A*SOD1 Mice Showed Altered Skeletal Muscle Mitochondrial Transport and Antioxidant Enzyme Protein Levels

**Figure 7.**
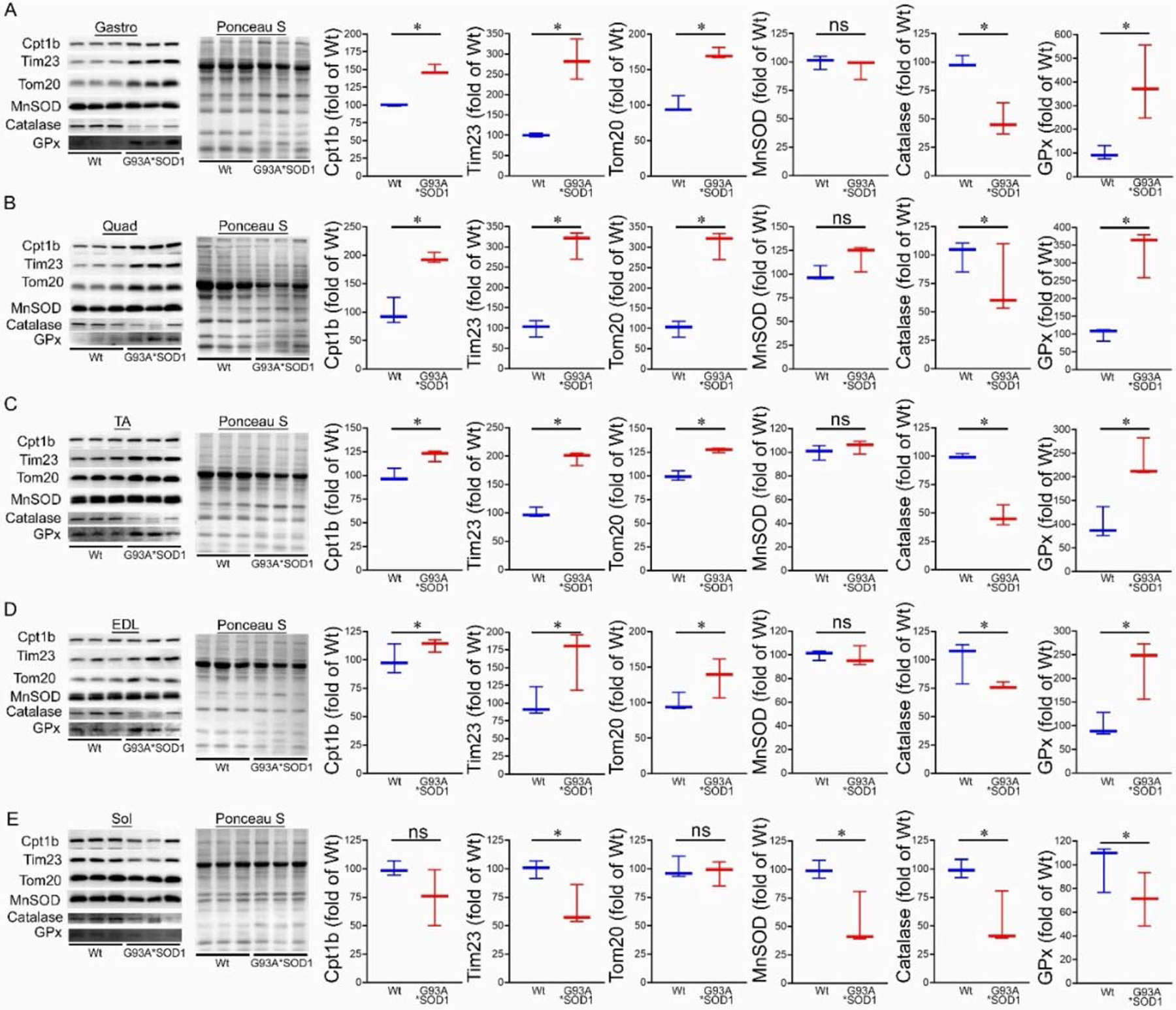
Altered levels of mitochondrial transport proteins and antioxidant enzymes proteins in G93A*SOD1 muscles. Representative Western blot images and densitometric quantification showing Tom 20, Tim 23, Cpt1b, MnSOD, Catalase, and GPx protein level in (**A**) Gastrocnemius (Gastro), (**B**) Quadriceps (Quad), (**C**) Tibialis Anterior (TA), (**D**) Extensor digitorum longus (EDL), and (**E**) Soleus (Sol) muscles (Wt and G93A*SOD1 mice). Ponceau S staining of the transfer membranes was used to confirm the approximately equal loading and transfer across the gel (n = 3 mice/group). Box plots showing the protein levels, boxes representing interquartile ranges, lines representing medians, and whiskers representing ranges. *p* < 0.05 between groups was considered statistically significant and was determined by the non-parametric Kruskal–Wallis test. * *p* < 0.05, ns = non-significant. Wt = Wildtype.

Under normal physiological conditions, mitochondrial proteins are synthesized in the cytosol and imported into the mitochondria by complex import machinery consisting of translocases of the outer mitochondrial membrane (Tom) and the inner mitochondrial protein (Tim). However, under pathological conditions, as observed with different mutant proteins associated with protein aggregation and human disease development, these aggregate proteins were reported to alter the mitochondrial import machinery by interacting with mitochondrial Tom 20 [27] and Tim 23 [22]. Therefore, we observed the protein level of Tom 20 and Tim 23 in the skeletal muscles (Gastro, Quad, TA, EDL, and Sol) isolated from Wt and G93A*SOD1 mice. Interestingly, we observed significantly increased Tom 20 and Tim 23 protein levels in the skeletal muscles of G93A*SOD1 mice compared to Wt mice (Figure 7A–E). Similarly, we also observed the increased protein levels of carnitine palmitoyl transferase 1b (Cpt1b), the key molecule in lipid metabolism facilitating the transport of medium-long chain fatty acids across the mitochondrial membrane to undergo *β*-oxidation (Figure 7A–E).

We also observed the protein levels of the principal antioxidant enzymes in the muscles, including superoxide dismutase (MnSOD), glutathione peroxidase (GPx), and catalase. Expression of mutant-SOD1 in all the muscles (Gastro, Quad, TA, EDL, and Sol) did not significantly affect the level of endogenous SOD1 (Figure 1F,H) and MnSOD in the G93A*SOD1 muscle compared to Wt muscles. However, significantly reduced catalase and increased GPx protein levels were observed in the skeletal muscle of the G93A*SOD1 mice compared to Wt mice (Figure 7A–E).

### 2.5. G93A*SOD1 Mice Skeletal Muscles Develop Mitochondrial Dysfunction

**Figure 8.**
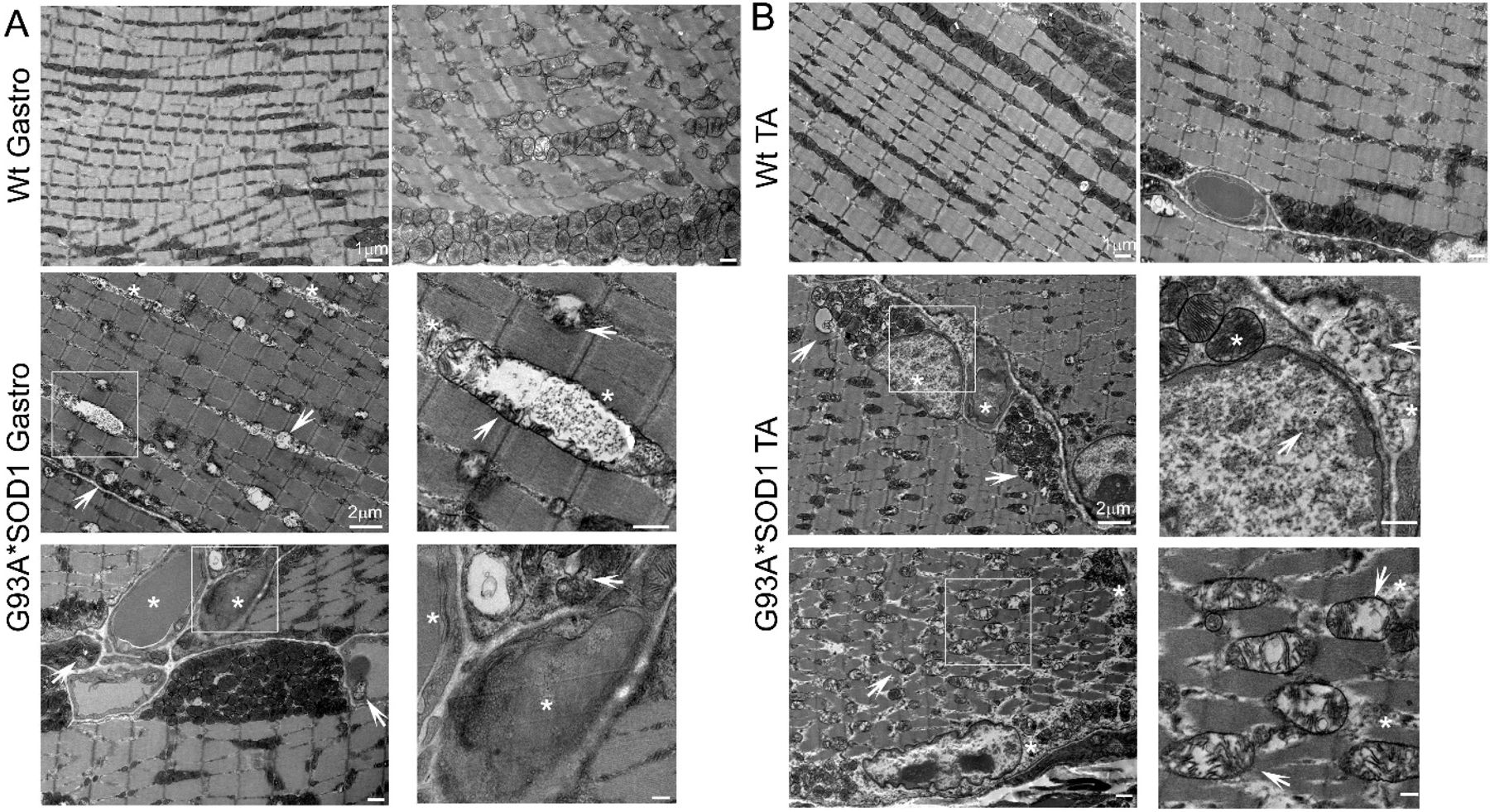
TEM showing abnormal mitochondria and protein aggregation in G93A*SOD1 muscles. Representative TEM images showing the ultrastructural organization of the (**A**) Gastrocnemius (Gastro) and (**B**) Tibialis Anterior (TA) muscle sections of Wt and G93A*SOD1 mice. All muscles examined were isolated from age-matched Wt and G93A*SOD1 mice (n = 2 mice/group). Asterisks (*) denote the presence of granulofilamentous material and electron-dense granular deposits in the subsarcolemmal region. White arrows depict vacuolized mitochondria with abnormal cristae structure. Scale bars: 1 μm and 2 μm.

Several studies proposed mitochondrial dysfunction as a contributing factor to ALS pathology. Studies demonstrating the pathology associated with mutant SOD1 also showed mutant SOD1 protein enrichment only in the mitochondria of affected tissues (specifically the spinal cord) of human, mouse, and rat models of ALS [28–30]. In addition, biochemical experiments using isolated mitochondria also reported a significant proportion of mutant SOD1 localization in the mitochondrial intermembrane space isolated from both the brain and liver of G93A*SOD1 mouse [30], yeast [31] and rat liver [32]. Therefore, we performed TEM analysis of the Gastro and TA muscle of the G93A*SOD1 and Wt mice to assess the ultrastructural changes associated with mutant-SOD1 expression in the muscle. We observed the accumulation of vacuolated, dilated, and disorganized mitochondria in the skeletal muscle of mutant-SOD1 mice (Figure 8A,B).

**Figure 9.**
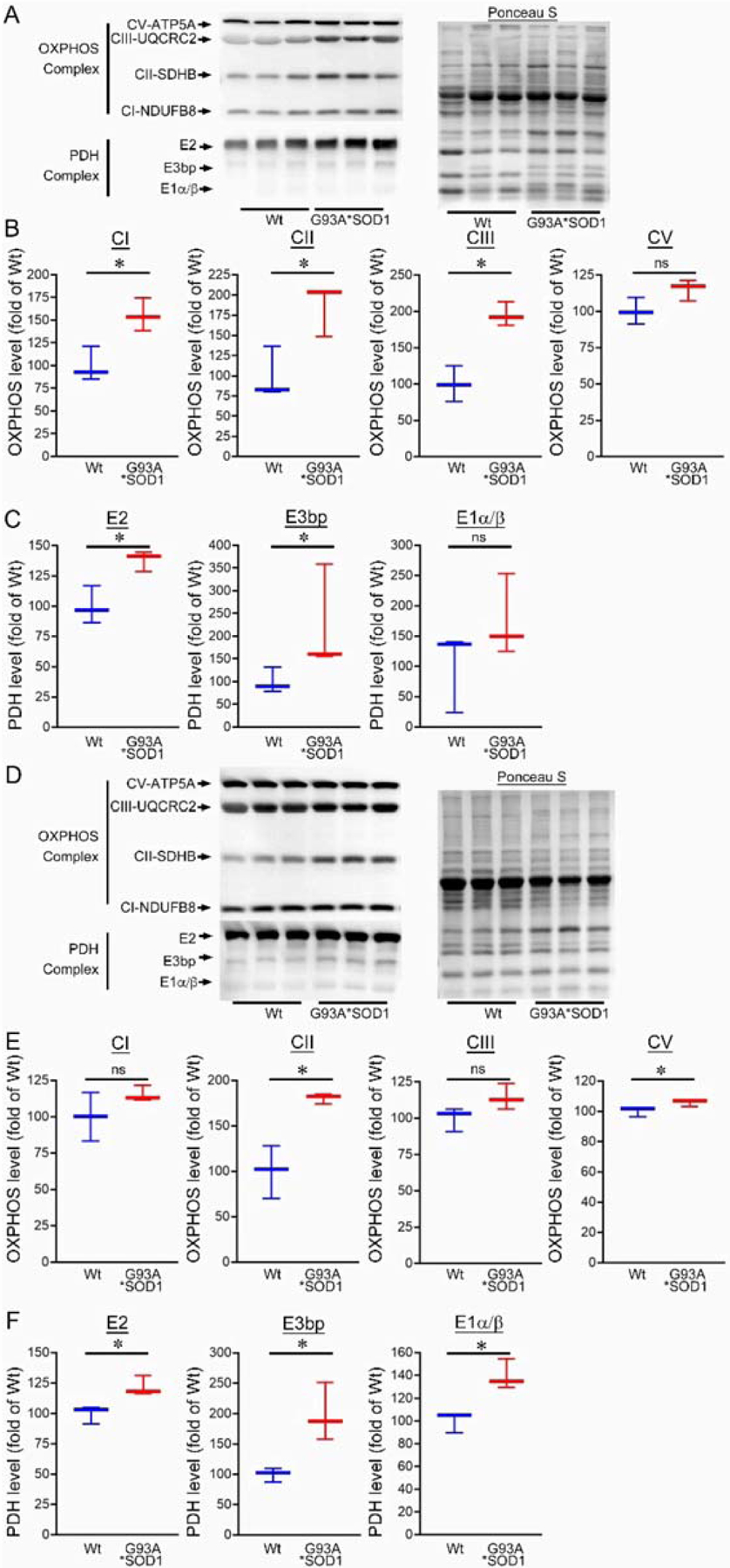
Altered mitochondrial OXPHOS and PDH complex protein levels in G93A*SOD1 skeletal muscles. Representative Western blot and densitometric quantification of the level of OXPHOS (Complex I, Complex II, Complex III, and Complex V) and PDH complex (E2, E3bp, and E1α/β) protein in the (**A**–**C**) Gastrocnemius (Gastro) and (**D**–**F**) Tibialis Anterior (TA) muscles (Wt and G93A*SOD1 mice). Ponceau S staining of the transfer membranes was used to confirm the approximately equal loading and transfer across the gel (n = 3 mice/group). Boxes represent interquartile ranges, lines represent medians, and whiskers represent ranges. *p* < 0.05 between groups was considered statistically significant and was determined by the non-parametric Kruskal–Wallis test. * *p* < 0.05, ns = non-significant. Wt = Wildtype, OXPHOS = oxidative phosphorylation, PDH = pyruvate dehydrogenase.

Ultrastructural analysis of the muscle also showed the presence of granulofilamentous material and electron-dense granular deposits in the subsarcolemmal region. We also measured the OXPHOS and PDH complex regulatory protein levels in Gastro and TA muscle isolated from G93A*SOD1 and Wt mice. Both the Gastro (Figure 9A–C) and TA (Figure 9D–F) muscles showed increased OXPHOS and PDH complex regulatory protein levels in G93A*SOD1 mice compared to Wt mice (Figure 9A,D).

**Figure 10.**
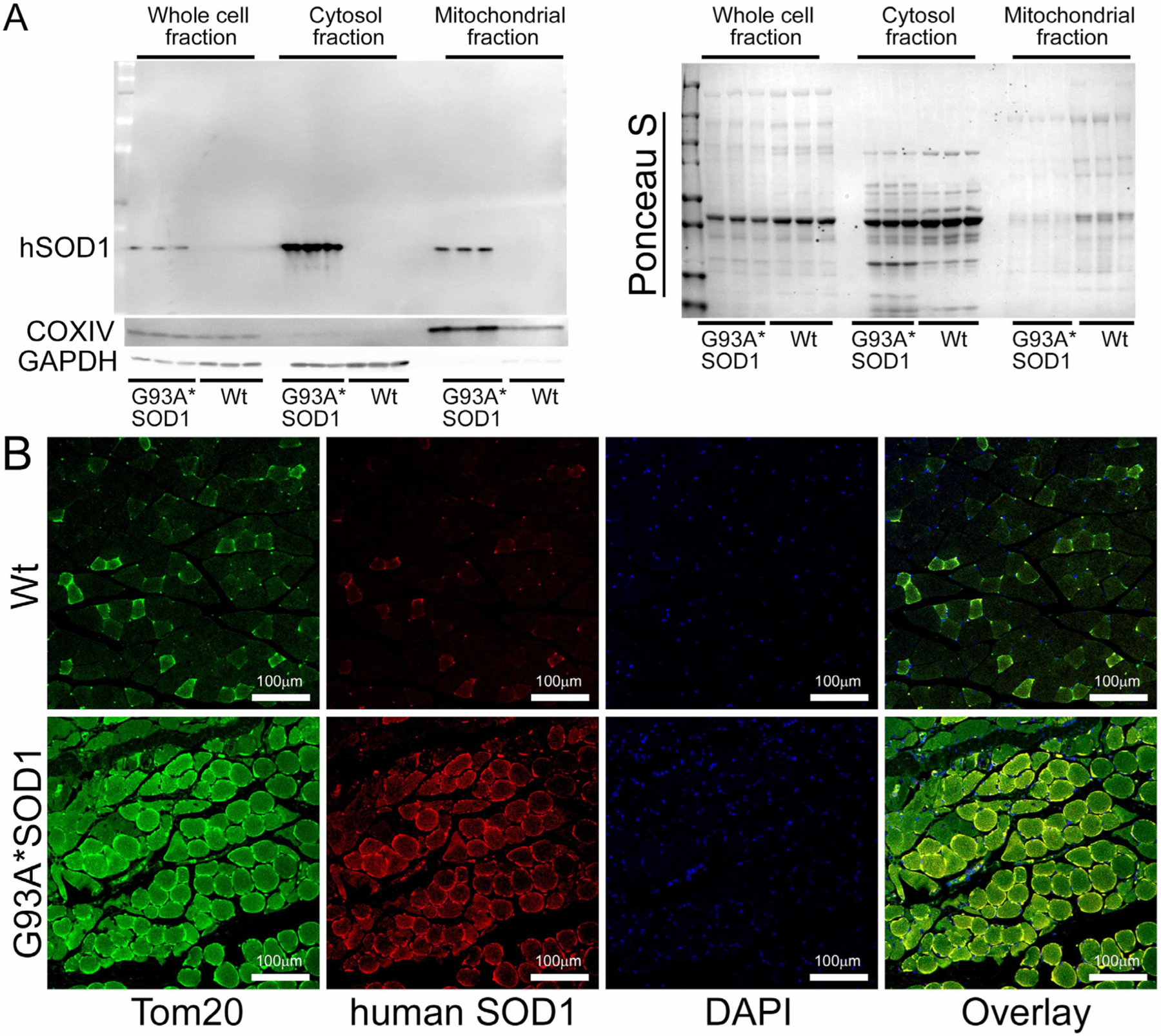
Mutant SOD1 mitochondrial localization in G93A*SOD1 skeletal muscles. (**A**) Western blot images showing the presence and partitioning of mutant SOD1 in the mitochondrial compartment of Gastro muscle tissue subcellular fractions consisting of the whole cell, cytosol, and mitochondrial fractions. Cytosolic protein GAPDH and mitochondrial membrane protein COXIV were used to assess the successful enrichment of the cytosol and mitochondria fractions. (n = 3 mice/group). Ponceau S staining of the transfer membranes was used to confirm the approximately equal loading and transfer across the gel. (**B**) Representative microscopic images showing anti-human SOD1 (red) and anti-Tom 20 (green) co-immunostaining in Gastro muscles. Scale bars: 100 μm. Wt = Wildtype.

We also observed mitochondrial localization of the mutant SOD1 using the isolated mitochondria from the Gastro muscle. Similar to the earlier reports in other organs, we found a proportion of the mutant SOD1 protein in the mitochondrial fraction (Figure 10A). We immunostained Gastro muscles with anti-human SOD1 antibody (in red) followed by co-immunostaining with mitochondrial outer membrane protein Tom 20 (in green), respectively. Interestingly, we observed colocalization of SOD1 with Tom 20 indicated by distinct yellow pixels (Figure 10B). Altogether, our mitochondrial fractionation and confocal immunofluorescence microscopic studies recapitulated a portion of mutant SOD1′s localization to mitochondria that were evident in the affected organs reported by earlier studies.

**Figure 11.**
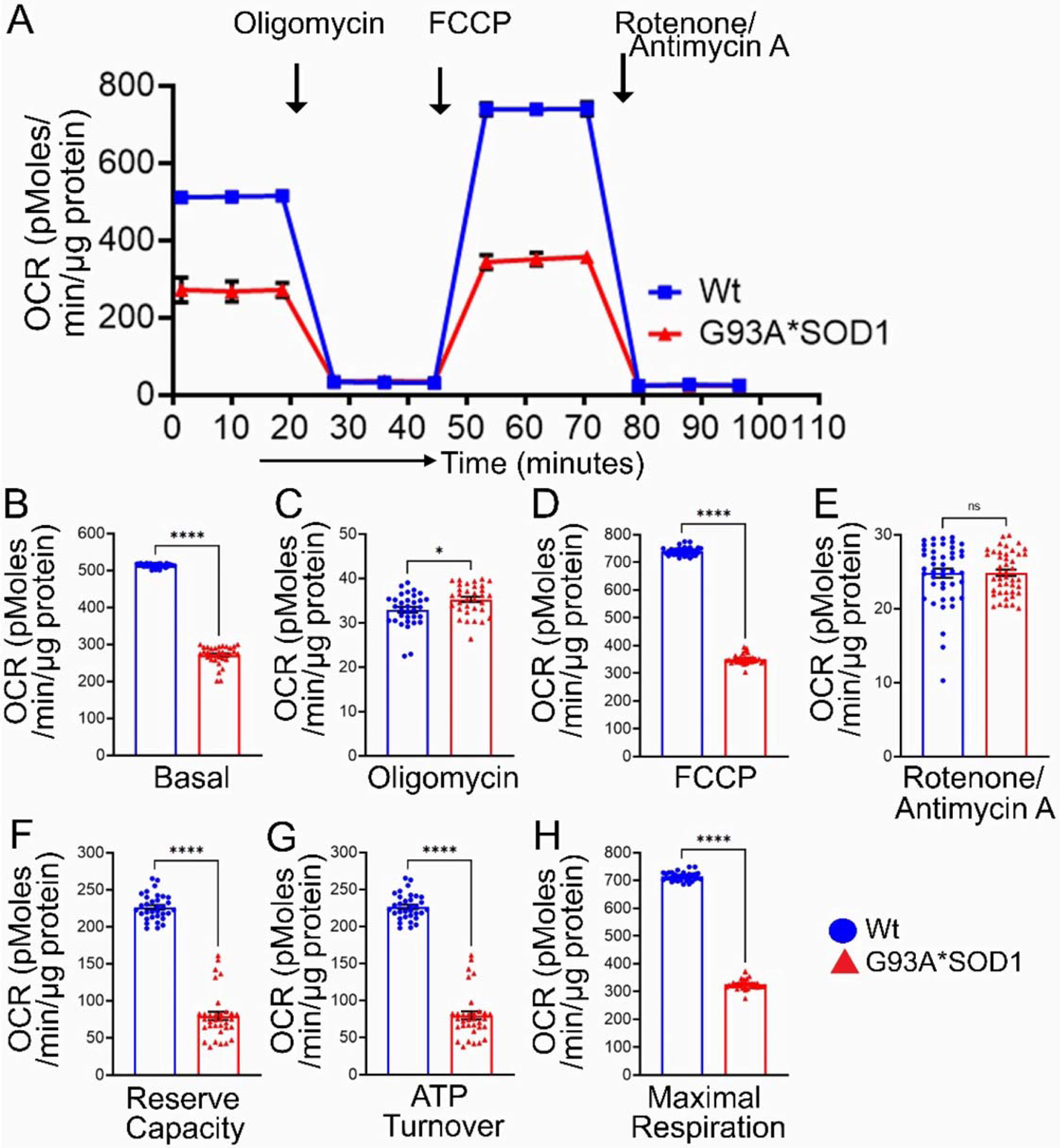
Reduced mitochondrial respiration in isolated mitochondria from G93A*SOD1 skeletal muscles. (**A**) Mitochondrial oxygen consumption rate (OCR) profiles in isolated Gastro muscle mitochondria of mice. Arrow indicates the sequential addition of oligomycin (1 μmol/L), carbonyl cyanide-4-phenylhydrazone (FCCP) (4 μmol/L), and rotenone (0.5 μmol/L) plus antimycin A (0.5 μmol/L). Each point represents an average OCR value, and OCR is expressed as picomoles O_2_/min/μg protein. Graphs showing OCR values at (**B**) baseline and after the addition of (**C**) oligomycin, (**D**) FCCP, and (**E**) rotenone plus antimycin A. Mitochondrial functional parameters calculated from the OCR values, including (**F**) reserve capacity, (**G**) ATP turnover, and (**H**) maximal respiration (n = 3 mice/group). Bars represent Mean± SEM, and the dots in the bars represent each data point in the experiment. *p* values were determined by unpaired Student’s *t*-test. * *p* < 0.05 and **** *p* < 0.0001; ns = nonsignificant. Wt = Wildtype.

Earlier studies reported defective mitochondrial respiration on the liver, brain, and spinal cord mitochondria of G93A*SOD1 mice using Clark-type electrode oxygraph [30]. For the mitochondrial functional and biochemical experiments, we used the Gastro muscle due to the larger size of the muscle. Real-time oxygen consumption rates (OCRs) in isolated mitochondria show a lower respiratory profile in the G93A*SOD1 muscle compared to the Wt muscle (Figure 11A). Basal respiration was significantly decreased in the G93A*SOD1 mitochondria indicating lower respiratory function compared to the Wt muscle (Figure 11B). The addition of oligomycin leads to a decrease in basal respiration, which indicates that oxygen consumption used to generate ATP was significantly increased in the G93A*SOD1 mitochondria (Figure 11C). The measurement of maximal OCR obtained by carbonyl cyanide-p-trifluoromethoxy-phenylhydrazone (FCCP) addition also showed a significant decrease in G93A*SOD1 mitochondria (Figure 11D). The non-mitochondrial OCR estimated by the addition of rotenone and antimycin A showed similar values in both G93A*SOD1 and Wt mitochondria (Figure 11E). Quantification of the respiratory reserve capacity (Figure 11F) and ATP turnover (Figure 11G) showed significantly decreased OCR values in G93A*SOD1 mitochondria. Finally, the quantified maximum respiration was also significantly lower in G93A*SOD1 mitochondria (Figure 11H). All these OCR data together suggested that mitochondria isolated from G93A*SOD1 muscle were functionally compromised.

## 3. Discussion

This study aimed to extensively characterize the expression pattern of mutant SOD1 protein and the associated skeletal muscles’ pathological sequelae resulting from the whole-body G93A*SOD1 expression in mice, including muscle morphology, function, histology, ultrastructure, and changes in signaling pathways. Despite extensive research on the G93A*SOD1 mouse model of ALS, most studies were designed to determine the role of motor neuron degeneration in the pathological with a superficial focus on their effect on muscle pathology. To date, a detailed histochemical, morphological, and ultrastructural analysis of the different skeletal muscles (Gastro, Quad, TA, Sol, and EDL) in G93A*SOD1 mice is still lacking. Nevertheless, several intriguing findings emerged from the study are as follows: (i) G93A*SOD1 mice developed muscle atrophy in all muscle (Gastro, Quad, TA, EDL, and Sol) fibers where the G93A*SOD1 protein is ubiquitously and ununiformly expressed; (ii) G93A*SOD1 mice had reduced endurance and exercise capacity compared to Wt mice; (iii) muscles of G93A*SOD1 mice showed an increased collagen deposition (Picro-Sirius Red staining), and chronic myopathic changes, including markedly increased fiber size variation, fibers with central nuclei, and endomysial fibrosis (WGA and H&E staining); (iv) Western blot analysis of the different muscles showed increased mitochondrial membrane proteins involved in mitochondrial protein transport and moderate altered levels of antioxidant enzyme protein; (v) ultrastructural analysis showed the presence of abnormal mitochondria with vacuolization and cristae disruption in Gastro and TA muscles of G93A*SOD1 mice; (vi) mitochondria isolated from Gastro muscles of G93A*SOD1 mice showed mitochondrial localization of a portion of mutant SOD1, reduced mitochondrial respiration, and altered level of OXPHOS and PDH complex protein. Overall, we characterized the skeletal muscle pathological sequelae resulting from the ubiquitous expression of human SOD1*G93A in mice.

Earlier studies showed that transgenic overexpression of both wildtype (*SOD1*^*Wt*^) and mutant SOD1 (G93A*SOD1) in mice significantly increased the superoxide-scavenging activity of SOD1 in different organs (skeletal muscle, neuronal tissues, liver, and kidney) [33]. Though an ideal control for the G93A*SOD1 mice would be a transgenic mouse overexpressing human wildtype *SOD1* (*SOD1*^*Wt*^), several studies reported that *SOD1*^*Wt*^ mice develop ALS-like phenotype and other neuronal dysfunction [34,35] and therefore, cannot be used as a control of mutant SOD1 experiments. In fact, studies suggested that misfolding of *SOD1*^*Wt*^ may also be responsible for developing ALS pathological processes regardless of SOD1 mutations [36]. All previous studies using SOD1*G93A overexpressing mice showed mutant-SOD1 levels in the skeletal muscle by qPCR and Western blot analysis. However, none of these previous studies characterized the nature of mutant SOD1 expression by histological staining. Therefore, our study is the first to demonstrate the nonuniform pattern of SOD1 expression in the skeletal muscle of these mice. As the overexpression of the mutant human SOD1 did not alter the level of endogenous mouse SOD1, we predict that the mutant SOD1 mediates all the phenotypes in this mouse. However, we don’t know what factors influence the expression of the mutant SOD1 in the muscle fiber and whether there is any preference for the muscle fiber type.

Despite extensive research over the years using rodent models of ALS having mutant SOD1, the exact nature of mutant SOD1-induced toxicity remains unknown, as the mutant action within different cells represented the onset and progression of the disease. Efforts to reduce mutant SOD1 expression in MNs by selective gene excision showed significant variability in the onset and early disease progression [37]. Surprisingly, the reduction of SOD1 protein and mRNA levels throughout the brain and spinal cord using antisense oligonucleotides to SOD1 in a rat model of ALS showed indistinguishable disease onsets and early disease. However, this study showed a slowed disease progression with a mild extension of survival to approximately ten days [38]. In contrast, the reduction of microglia’s mutant protein level showed little effect on the early disease phase but sharply slowed later disease progression [37]. Similarly, reducing mutant SOD1 (SOD1^G37R^) mRNA and protein by gene excision in skeletal muscle could not preserve grip strength, rescue survival, and affect disease onset in mice [39]. On the contrary, restricted expression of mutant SOD1 in the skeletal muscle of mice at a similar level as in the G93A*SOD1 mice developed muscle atrophy with a reduced tetanic and specific force, NMJ abnormalities, and an MN distal axonopathy [16–18]. Surprisingly, all these studies focusing on the skeletal muscle phenotype lack a detailed histological and morphological analysis of the muscles. Nevertheless, these results highlighted the importance of identifying the mechanisms involved in skeletal muscle pathology and its contribution to the development of ALS pathobiology.

In the present study, all the muscles of G93A*SOD1 mice showed the activation of the NF-κB-MuRF1 signaling axis. Furthermore, muscle-specific activation of NF-κB was reported to cause profound muscle wasting through the increased expression of MuRF1 in skeletal muscle [22,28,40,41]. Despite all these studies, how NF-κB activation and modulation of inflammation could lead to MN death and muscle pathology in G93A*SOD1 mice remain unknown. Both human and mouse models of ALS showed activation of NF-κB in different types of cells, including microglia, astrocyte, and neuron [42]. Surprisingly, astrocyte NF-κB inhibition could not confer neuroprotection in vitro or in vivo in the G93A*SOD1 mice [42,43]. In contrast, NF-κB activation in microglia by transgenic overexpression was sufficient to increase MN impairment and dysfunction, shortening the overall survival in G93A*SOD1 mice [42]. NF-κB suppression in microglia showed modest beneficial effects in the G93A*SOD1 mice, significantly delaying the disease progression and increasing survival by 20 days [42]. However, NF-κB activation in astrocytes in the pre-symptomatic phase of ALS triggered a beneficial microglia activation (indicated by decreasing disease markers, delaying loss of MNs and denervation, and prolonging the pre-symptomatic phase) and slowed disease progression in G93A*SOD1 mice [44]. Selective inhibition of neuronal NF-κB activation also resulted in neuroprotection in G93A*SOD1 mice with a reduction of mutant SOD1 accumulations and a moderate extension of median survival by 15 days [45]. However, the skeletal muscle pathology associated with the NF-κB activation/inhibition in these studies was overlooked. Therefore, more studies are required to determine the role of muscle fiber-specific NF-κB activation in the development and progression of skeletal muscle pathology observed in these ALS mice models. In addition to the NF-κB-MuRF1 signaling axis, several other mechanisms and the signaling axes may be responsible for muscle atrophy, resulting in significant muscle weight loss in these mice. Therefore, future studies should be directed toward determining the mechanisms of muscle atrophy in these mice.

Several earlier studies demonstrated mitochondrial dysfunction as a significant contributor to the development of ALS pathology. Mice and rat models of ALS and postmortem tissue samples from ALS patients showed the mutant SOD1 protein enrichment selectively in the mitochondria derived from the affected tissues (specifically the spinal cord) [28–30]. However, a series of biochemical experiments in isolated, purified brain and liver mitochondria showed a significant proportion of enzymatically active hSOD1 (~0.5–2% of total cellular hSOD1) localized in the mitochondrial intermembrane space of mitochondria of G93A*SOD1 mouse [30]. Similarly, fractions of active mutant SOD1 were also reported to localize within the mitochondrial intermembrane space of yeast [31] and rat liver [32]. Electron microscopic examination also confirmed the presence of mutant SOD1 within spinal cord mitochondria [46,47]. Therefore, mitochondrial dysfunction within MNs considered an early pathological feature of ALS pathology in G93A*SOD1 mice [11]. In fact, we also observed the presence of vacuolized, irregularly shaped giant mitochondria, mutant SOD1 protein in the mitochondrial fraction, defects in mitochondrial respiration, and altered OXPHOX and PDH regulatory protein levels in the skeletal muscle of the G93A*SOD1 mouse. In association with this, we observed an increased mitochondrial outer and inner membrane transport proteins (Tom 20 and Tim 23) in the skeletal muscles of G93A*SOD1 mice. Therefore, the mutant SOD1 may form aggregates onto mitochondrial integral membrane components of import machinery, impeding mitochondria’s normal functioning. Further studies are required to identify the tissue-specific factors that modify mitochondrial import of the aggregate proteins and how this pathway contributes to the mechanisms of altered muscle metabolism and skeletal muscle pathology in these mice.

## 4. Material and Methods

### 4.1. Materials

The materials used are as follows: Cell Lytic M (C2978, Sigma-Aldrich; St. Louis, MO, USA), Complete Protease Inhibitor Cocktail (Roche; Basel, Switzerland), pre-cast 7.5–15% Criterion Gels (BioRad; Hercules, CA, USA), Oligomycin (Sigma-Aldrich; St. Louis, MO, USA), FCCP (Sigma-Aldrich; St. Louis, MO, USA), Rotenone (Sigma-Aldrich; St. Louis, MO, USA), Antimycin A (Sigma-Aldrich; St. Louis, MO, USA), Ponceau S (Acros Organic; Geel, Belgium), and Vectashield Hardset (Vector Labs, H1400; Burlingame, CA, USA).

### 4.2. Animals

We used 100-day-old Wt (G93A*SOD1 Non-carrier) and G93A*SOD1 mice on C57BL/6 background strain for all our experiments. We obtained both G93A*SOD1 and their Wt control mice from Jackson Laboratories (Bar Harbour, ME, USA) [16,48]. We used both male and female mice for our experiments for both genotypes. The mice were housed in a well-regulated environment in properly maintained cages with water and a regular chow diet ad libitum, following a 12 h light-dark cycle. Animals were handled following the procedures in compliance with the Guide for the Care and Use of Laboratory Animals [49] and the animal protocol approved by the Animal Care and Use Committee of LSU Health-Shreveport. In addition, the care of animals was taken following the NIH Guide for the Care and Use of Laboratory Animals.

### 4.3. Muscle Endurance Capacity

Grip strength measurements were used to evaluate skeletal muscle endurance capacity. The forelimb grip strength of the mice (Wt and G93A*SOD1) was assessed, as described previously [50–52]. Briefly, we acclimated the mice for at least 10 min before the experiment. Following acclimation, mice were placed on the mesh grid attached to the grip strength meter (1027SM; Columbus Instruments, Columbus, OH, USA), allowed to hold the grid using their forelimbs, and gently pulled the tail of the mice away from the grid to the point when the mice released the hold of the grid. The gauge measures the tension produced at the point of release. Following each measurement, the mouse rested for 1 min, and then the process was repeated for up to five trials per mouse. The data for each trial for every mouse was analyzed using the manufacturer’s software for the grip strength meter.

### 4.4. Muscle Exercise Tolerance

Exercise tolerance in mice (Wt and G93A*SOD1) were assessed by a graded maximal exercise test, as described previously [52,53]. Briefly, mice were acclimated to the treadmill (OxyletPro, Panlab; Harvard Apparatus, Holliston, MA, USA) in three training sessions and allowed to rest for one week before the experiment. For acclimation, mice were placed in the motionless treadmill, followed by activation of the shock grid (1.5 mA). Subsequently, the treadmill was set up to a walking speed of 6 m/minute (m/min) for 5 min and progressively increased the speed to 12 m/min for 12 min. After a week of rest following acclimation, mice were placed on the treadmill at 0^0^ incline and activated the shock grid. The mice were allowed to run on the treadmill with progressively increasing speeds and inclinations until exhaustion [52,53]. Mice spending more than 5 s on the shock grid or getting more than 10 shocks indicate exhaustion time. The data were analyzed using Metabolism version 3.0 software (Harvard Apparatus).

### 4.5. Muscle Isolation and Morphometry

To evaluate muscle morphometry, we subjected mice (Wt and G93A*SOD1) to isoflurane-induced anesthesia and isolated five different muscles (Gastro, Quad, TA, EDL, and Sol) from both limbs [52]. These muscle tissues were then cleaned to remove any extra tissue and processed as per the experimental requirements. For muscle morphometry, the cleaned muscle tissues were washed with 1X phosphate-buffered saline (PBS) to remove any extra fur, fat, or bloodstains and then removed excess PBS. Tissue weights were measured using an OHAUS electronic balance (OHAUS Corporation, Parsippany, NJ, USA).

### 4.6. Protein Isolation and Western Blotting

We prepared total protein from the different muscles of mice (Wt and G93A*SOD1) with the Cell Lytic M (Sigma-Aldrich, St. Louis, MO, USA) lysis buffer containing Complete Protease Inhibitor Cocktail (Roche, Basel, Switzerland), as described previously [54,55]. For muscle lysis, we homogenized all the muscles twice using a bead homogenizer followed by sonication. Subsequently, we centrifuged the lysed muscle homogenates at 12,000× *g* for 15 min to sediment the insoluble cell debris and used soluble supernatant for our further steps. Following centrifugation, the protein concentration was measured using the Bradford reagent (Bio-Rad, Hercules, CA, USA) using the bovine serum albumin (BSA) (Bio-Rad) as the protein standard. The prepared protein samples were subsequently subjected to SDS-PAGE, transferred to polyvinylidene difluoride (PVDF) membranes (Bio-Rad), blocked, and incubated with primary antibodies. Finally, the membranes were subsequently incubated with alkaline phosphatase-conjugated secondary antibodies (Jackson ImmunoResearch Laboratories, Inc., West Grove, PA, USA), developed with ECF reagent (Amersham, Amersham, UK), and imaged with Chemidoc Touch Imaging System (Bio-Rad). Densitometric analysis of protein bands performed by using ImageJ software version 1.53q (NIH, Bethesda, MD, USA). The primary antibodies used in this manuscript are muscle RING-finger protein-1 (MuRF1; 1:200; sc-398608; Santa Cruz Biotechnology), SOD1 (1:500; 37385; Cell Signaling, Danvers, MA, USA), pNFkB (1:500; 3033; Cell Signaling), Total NFkB (1:1000; 8242; Cell Signaling), Cpt1b (1:500; PA5–79065; Invitrogen, Waltham, MA, USA), Tim 23 (1:200; sc-514463; Santa Cruz Biotechnology), Tom 20 (1:200; sc-11415; Santa Cruz Biotechnology), MnSOD (1:500; 06984; Millipore, Burlington, MA, USA), Catalase (1:500; 129805; Cell Signaling), Glutathione Peroxidase 1(GPX1; 1:500; ab108427; Abcam, Cambridge, UK), OxPhos (1:1000; MS604; Abcam), PDH (1:1000; ab110406; Abcam), GAPDH (1:10000; MAB374; Millipore), and COXIV (1:2500; 4844; Cell Signaling).

### 4.7. Histological Analyses

For the histological analysis of the muscle sections, we used serial sections of 5μm cut from paraffin-embedded blocks of muscles (Wt and G93A*SOD1 mice). We deparaffinized and hydrated the muscle sections, followed by staining with Picro-Sirius Red [52,54–56] and H&E (H&E staining kit; H3502; Vector Laboratories) using the manufacturer’s protocol. In bright-field mode, stained slides were imaged in an investigator-blinded manner using Olympus BX40 microscope (Tokyo, Japan). Subsequently, we quantified the collagen deposition in the muscle sections using NIH ImageJ software as previously described [56]. Briefly, we quantified the red-stained and non-stained myocyte areas in each image section using color-based thresholding. The amount of collagen deposition as the percentage of the red-stained area was quantitated. H&E staining showed the presence of central nuclei and nuclei infiltration in different muscle sections (Wt and G93A*SOD1 mice).

### 4.8. WGA Staining

Wheat germ agglutinin (WGA) staining was used to evaluate the cross-sectional area (CSA) of the myofibers [52,55,56]. Formalin-fixed paraffin-embedded tissue blocks were cut into thin serial sections, deparaffinized, subjected to antigen retrieval (by boiling at 100 °C in 10 mmol/L sodium citrate buffer (pH 6.0)), blocked (1% bovine serum albumin, 0.1% cold water fish skin gelatin, and 1% Tween 20 in PBS), and incubated with Alexa Fluor 488 wheat germ agglutinin (5 μg/mL; Invitrogen). DAPI (Invitrogen) was used to counterstain nuclei and mounted using Vectashield Hardset antifade mounting media for fluorescence (Vector Laboratories). Stained sections were imaged in an investigator-blinded manner using Nikon A1R high-resolution confocal microscope (Nikon Instruments Inc., Melville, NY, USA).

### 4.9. Immunohistochemistry

Immunohistochemistry of mice muscle sections (Wt and G93A*SOD1) for SOD1 was used to observe the expression pattern of mutant-SOD1 in muscle sections, as described previously [55]. Briefly, d thin serial sections of 5 μm from paraffin-embedded tissues were subjected to subsequent deparaffinization, hydration, antigen retrieval (H-3300, Vector Laboratories, Burlingame, CA, USA), blocking of endogenous peroxidases (0.3% *v*/*v* hydrogen peroxide; Bloxall, SP-6000, Vector Laboratories, Burlingame, CA, USA), blocking with 5% serum (Vector Laboratories, Burlingame, CA, USA), and primary antibody incubation in blocking serum overnight. The sections were incubated with secondary antibody (Vector Laboratories, Burlingame, CA, USA) amplified antigen-antibody interaction using VECTASTAIN Elite ABC Peroxidase kit (PK-6100, Vector Laboratories, Burlingame, CA, USA) and visualized using DAB-chromogen System (SK-4105, Vector Laboratories, Burlingame, CA, USA). Subsequently, the sections were counterstained with hematoxylin, mounted using Cytoseal XYL mounting medium (Thermo Scientific), and imaged using Olympus BX40 microscope (Olympus Life Science, Waltham, MA, USA). The primary antibody used in this experiment is SOD1 (1:100; 37385; Cell Signaling).

### 4.10. Transmission Electron Microscopy

We used Gastro and TA muscles from Wt and G93A*SOD1 mice to observe the ultrastructural changes. Muscles were cut into small cubes and subsequently fixed in glutaraldehyde-cacodylate-OsO_4_ buffer, counterstained (with uranyl acetate and lead salts). Finally, the sections were imaged using a JEOL JEM-1400 transmission electron microscope (JEOL, Peabody, MA, USA).

### 4.11. Immunostaining

To assess the mitochondrial localization of SOD1 in the muscle fibers, we employed immunostaining of gastrocnemius tissue sections as described previously [52,55,56]. Briefly, we used 5 μm serial sections from paraffin-embedded blocks. Subsequently, we subjected the cut serial sections to deparaffinization, hydration, antigen retrieval (by boiling at 100 °C in 10 mmol/L sodium citrate buffer (pH 6.0)), enhancer, and blocking (1% bovine serum albumin, 0.1% cold water fish skin gelatin, and 1% Tween 20 in PBS). Following blocking, the sections were incubated with primary antibodies, Alexa Fluor conjugated dyes, and DAPI. Sections were mounted using Vectashield Hardset antifade mounting media imaged using Nikon A1R high-resolution confocal microscope in an investigator-blinded manner. The primary antibodies and secondary used in this experiment were SOD1 (1:100; 37385; Cell Signaling), TOM 20 (1:100; ab56783; Abcam), Alexa Fluor 488 (A11034; Invitrogen), and Alexa Fluor 568 (A11031; Invitrogen).

### 4.12. Mitochondrial Isolation and Respiration Assessment

We isolated mitochondria from freshly isolated Gastro muscles from both Wt and G93A*SOD1 mice to examine mitochondrial bioenergetics changes. Using mannitol-sucrose-ethylene glycol tetraacetic acid (EGTA) buffer containing 225 mM mannitol, 75 mM sucrose, 5 mM HEPES, and 1 mM EGTA (pH 7.4), we homogenized isolated muscle tissue with a glass /Teflon Potter Elvehjem homogenizer as described previously [55–57]. The homogenized muscle lysates were subjected to differential centrifugation to obtain mitochondrial fraction. We used these fresh mitochondrial fractions to assess mitochondrial oxygen consumption rates (OCR) using a Seahorse XFe24 Extracellular Flux Analyzer (Agilent, Santa Clara, CA, USA). Isolated mitochondria (100 μg protein per well) were seeded in Xfe24 Seahorse plates containing respiration media (220 mM mannitol, 70 mM sucrose, 10 mM KH_2_PO_4_, 5 mM MgCl_2_, 2 mM HEPES, 1 mM EGTA, 0.2% *w*/*v* fatty acid-free bovine serum albumin, 7 mM pyruvate, and 1 mM malate; pH 7.4). Mitochondrial OCR was recorded with the sequential addition of 1 μM oligomycin, 4 μM carbonyl cyanide p-(trifluromethoxy) phenyl-hydrazone (FCCP), and 0.5 μM rotenone plus 0.5 μM antimycin A.

### 4.13. Statistical Analysis and Reproducibility

All animal studies were performed in an investigator-blinded manner when possible. GraphPad Prism software (v8.4.1, La Jolla, CA, USA) was used for all statistical analyses. We analyzed the data using a two-tailed, unpaired Student’s *t*-test, followed by Tukey’s post hoc test. We used the Kruskal–Wallis test for certain data sets with smaller sample sizes. Data were presented in graphs showing median and interquartile ranges as well as dots. Statistical significance was determined by *p*-values less than 0.05.

## 5. Conclusions

In conclusion, this study reported, for the first time, a comprehensive skeletal muscle pathological sequelae resulting from ubiquitous and nonuniform expression of mutant SOD1 in the skeletal muscles of G93A*SOD1 mice. All muscle fibers (Gastro, Quad, TA, EDL, and Sol) in G93A*SOD1 mice developed muscle atrophy and signs of chronic myopathy, including fibrosis, fiber size variation, and signs of inflammation. In association with this muscle histopathology, muscles of G93A*SOD1 mice also showed increased mitochondrial membrane proteins involved in protein transport and modest alterations in the levels of antioxidant enzyme proteins. Molecular and biochemical characterization of the G93A*SOD1 mice muscle mitochondria showed the presence of abnormal mitochondria, defects in mitochondrial respiration, and altered levels of OXPHOS and PDH complex protein. A portion of human mutated-SOD1 protein localized in muscle mitochondria may also contribute to the observed mitochondrial dysfunctions. Mitochondrial defects and dysfunction likely contribute to skeletal muscle dysfunction in these mice. The precise pathogenic role of mutated SOD1 and the molecular mechanism of mitochondrial dysfunction in the skeletal muscle mice models of ALS can help to identify new effective therapeutic strategies. Therefore, findings from our study suggest a direct pathological role of mutant SOD1 in the skeletal muscle of G93A*SOD1 mice contributing to the reported ALS pathobiology.

## Data Availability

All datasets generated and analyzed during the current study are available from the corresponding author on reasonable request.
